# Assessing the potential for up‐cycling recovered resources from anaerobic digestion through microbial protein production

**DOI:** 10.1111/1751-7915.13600

**Published:** 2020-06-11

**Authors:** Kristof Verbeeck, Jo De Vrieze, Ilje Pikaar, Willy Verstraete, Korneel Rabaey

**Affiliations:** ^1^ Center for Microbial Ecology & Technology (CMET) Ghent University Coupure Links 653 Gent B‐9000 Belgium; ^2^ ArcelorMittal Belgium John F. Kennedylaan 51 B‐9042 Gent Belgium; ^3^ Centre for Advanced Process Technology for Urban Resource recovery (CAPTURE); ^4^ Advanced Water Management Centre (AWMC) The University of Queensland St Lucia Qld 4072 Australia; ^5^ Avecom NV Industrieweg 122P Wondelgem B‐9032 Belgium

## Abstract

Anaerobic digesters produce biogas, a mixture of predominantly CH_4_ and CO_2_, which is typically incinerated to recover electrical and/or thermal energy. In a context of circular economy, the CH_4_ and CO_2_ could be used as chemical feedstock in combination with ammonium from the digestate. Their combination into protein‐rich bacterial, used as animal feed additive, could contribute to the ever growing global demand for nutritive protein sources and improve the overall nitrogen efficiency of the current agro‐ feed/food chain. In this concept, renewable CH_4_ and H_2_ can serve as carbon‐neutral energy sources for the production of protein‐rich cellular biomass, while assimilating and upgrading recovered ammonia from the digestate. This study evaluated the potential of producing sustainable high‐quality protein additives in a decentralized way through coupling anaerobic digestion and microbial protein production using methanotrophic and hydrogenotrophic bacteria in an on‐farm bioreactor. We show that a practical case digester handling liquid piggery manure, of which the energy content is supplemented for 30% with co‐substrates, provides sufficient biogas to allow the subsequent microbial protein as feed production for about 37% of the number of pigs from which the manure was derived. Overall, producing microbial protein on the farm from available methane and ammonia liberated by anaerobic digesters treating manure appears economically and technically feasible within the current range of market prices existing for high‐quality protein. The case of producing biomethane for grid injection and upgrading the CO_2_ with electrolytic hydrogen to microbial protein by means of hydrogen‐oxidizing bacteria was also examined but found less attractive at the current production prices of renewable hydrogen. Our calculations show that this route is only of commercial interest if the protein value equals the value of high‐value protein additives like fishmeal and if the avoided costs for nutrient removal from the digestate are taken into consideration.

## Graphical abstract

Anaerobic digesters are the core engine for a biorefinery producing microbial protein. Either the biogas can be used directly for its production, or via biogas upgrading the separate CO_2_ can be combined with electrolytic hydrogen to produce more protein.

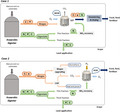

## Introduction

Anaerobic digestion (AD) is a mature and energy‐efficient technology, able to convert a broad variety of organic (waste) streams into biogas, a renewable source of methane (CH_4_), and digestate, a nutrient‐rich organic residue (Appels *et al*., 2011). The AD process has successfully been put forward as the first commercial ‘waste‐to‐energy’ bioreactor technology dealing with low‐value carbon‐rich waste streams, like manure, and is often envisaged as one of the key low‐carbon technologies in the decarbonized energy mix of the future (Kampman *et al*., 2016). Today, 70 % of the more than 17 000 AD plants in the European Union are running on agricultural streams, with in many cases manure as the primary feedstock, and often a second substrate, for example grass or corn (typical on‐farm feedstock), or various off‐site feedstock, such as slaughterhouse waste, fats and organic household waste, to increase the biogas production and operational stability of the process (EBA, 2017). Biogas is typically valorized (and incentivized) through the production of electricity in a combined heat and power (CHP) unit, but recently, a study pointed out that the inherently low value of methane as energy carrier can be bypassed if the methane is considered as a renewable C_1_ feedstock for the production of bio‐based chemicals from industrial waste CO_2_ and grid‐injected biomethane (Verbeeck *et al*., 2018). The conceptual idea to couple anaerobic digesters to centralized chemical industries *via* the existing natural gas grid, valorizing renewable methane as a green carbon source in production processes, has opened new utilization options for the biogas industry, potentially even without being reliant on legal support schemes to guarantee a profitable investment (Verbeeck *et al*., 2018).

In addition to the conversion of biomass to biogas, anaerobic digesters are excellent liberators of ammonia and phosphates from the complex feedstock. Manure represents an exquisite mining resource, with typical concentrations ranging between 2.1–6.7 g N L^−1^ and 0.2–1.6 g P L^−1^ in piggery waste (Pintucci *et al*., 2017). At a yearly mass flow of 1.3–1.8 billion tons of livestock manure in the EU alone (Foged *et al*., 2012), manure represents one of the largest secondary flow of nutrients through agricultural supply chains. Historically, digestate produced from the process has been applied to land as an organic fertilizer or soil conditioner, enabling local nutrient cycling. The application to agricultural land is today often limited, due to legislative restrictions on nutrient application of digestate for agricultural purposes in areas with nutrient surpluses (Coppens *et al*., 2016). Due to a growing awareness of the economic and environmental costs incurred with the inefficient use of mineral fertilizers in current agricultural plant and meat production, technologies to recover nitrogen and phosphorus from used water have gained more attention in recent years, preventing excessive losses of phosphates and reactive nitrogen species (NH_4_^+^, NO_2_^−^, NO_3_^−^) into our biosphere (Verstraete *et al*., 2016). Approaches such as ammonia stripping (Pedizzi *et al*., 2017), electrochemical ammonium extraction (Desloover *et al*., 2012; Desloover *et al*., 2015) and struvite precipitation (Le Corre *et al*., 2009) are some of the key systems to directly refine and recover nutrients from anaerobic digestate, and produce a marketable product. However, the fertilizer products typically derived from digestate (like (NH_4_)_2_SO_4_, NH_4_OH and struvite) achieve, at present, a market value not higher than 20% of their intrinsic value, because they are endowed with an irregular composition, limited supply quantities and a poor physical condition. Revenues can only slightly compensate the investment and running costs incurred with the transportation, treatment or upgrading efforts (De Vrieze *et al*., 2019). Today, ammonia–nitrogen in digestate streams is, thus, mainly destroyed through biological nitrogen removal processes (nitrification–denitrification or partial nitritation–annamox), rather than recovered and reused (Matassa *et al*., 2015). To ensure more secure and sustainable markets for recovered nutrients, with a lower dependence on land application, novel and higher‐value products need to be created. The integration of technologies to upgrade low‐value raw recovered nutrients to high‐value end‐products will be a key feature of next‐generation AD installations.

Recently, innovative approaches implementing bacteria to produce microbial protein (MP), also known as single‐cell protein (SCP), within an AD context have been proposed (Matassa *et al*., 2015). This MP is a more resource‐efficient and high‐rate protein that is put forward as a viable alternative for the conventional agricultural‐based protein production chain, which is rather inefficient when it comes to the use of reactive nitrogen, and which causes serious environmental damages (Galloway *et al*., 2014; Steffen *et al*., 2015). Interestingly, MP can be aerobically produced from renewable raw materials, like NH_3_, CH_4_, CO_2_ and H_2,_ generating a sustainable protein‐rich biomass that can be used as a fertilizer, feed or food additive (Pikaar *et al*., 2018a). Anaerobic digesters are providers of the most important building blocks for MP biosynthesis: carbon, energy (chemical or electrical) and NH_3_ are available at considerably large amounts.

The idea to utilize biogas as a local source of CH_4_ for MP production by methane‐oxidizing (methanotrophic) bacteria (MOB) has gained renewed interest (Pieja *et al*., 2017; Steinberg *et al*., 2017), mainly due to the pressing need to find new business models for AD biorefinery concepts, and the successful market entry of two natural gas based MP production facilities using MOB (UniBio A/S and Calysta) (Ritala *et al*., 2017). Methanotrophs grow on methane as their sole carbon and energy source, directly converting methane into bacterial biomass, while assimilating mineral nitrogen (*i.e*. ammonium) into high‐quality protein. The end‐products of this MP production technology have been approved as protein‐rich feed additive, having an amino acid profile close to high‐quality animal protein (Øverland *et al*., 2010). As an alternative to MOB, autotrophic hydrogen‐oxidizing bacteria have recently received attention as potential production strains, due to their unique metabolic ability to fix CO_2_ into new cellular material, using H_2_ and O_2_ as electron donor and electron acceptor respectively. The HOB can contain up to 75% crude protein (12% N) based on cell dry weight (CDW), which is much higher than the 50, 46 and 15% protein content in yeast, soybean and wheat grain respectively (Matassa *et al*., 2016b). The fact that HOB can be grown on recovered CO_2_, electrolytically produced H_2_ and O_2_, and recovered NH_3_ can potentially create effective niches for novel application in the context of resource recycling and upgrade, mainly because HOB can exploit the potential of renewable energy generation to capture CO_2_ from point sources (Pikaar *et al*., 2017). Carbon feedstocks under consideration for MP production in an AD context include CH_4_ from biogas, CO_2_ collected from the process of upgrading biogas to biomethane, or the CO_2_ emissions coming from the biogas combustion in an on‐site cogeneration unit. The concept that through solar power, coupled to electrolytic H_2_ production, reactive nitrogen in the form of ammonia present in anaerobic digestate can be upgraded to valuable feed protein, thereby shortcutting current protein production processes, opens new options for anaerobic digestion as important driver of an entirely new decentralized economy for sustainable on‐site feed production.

**Fig. 1:**
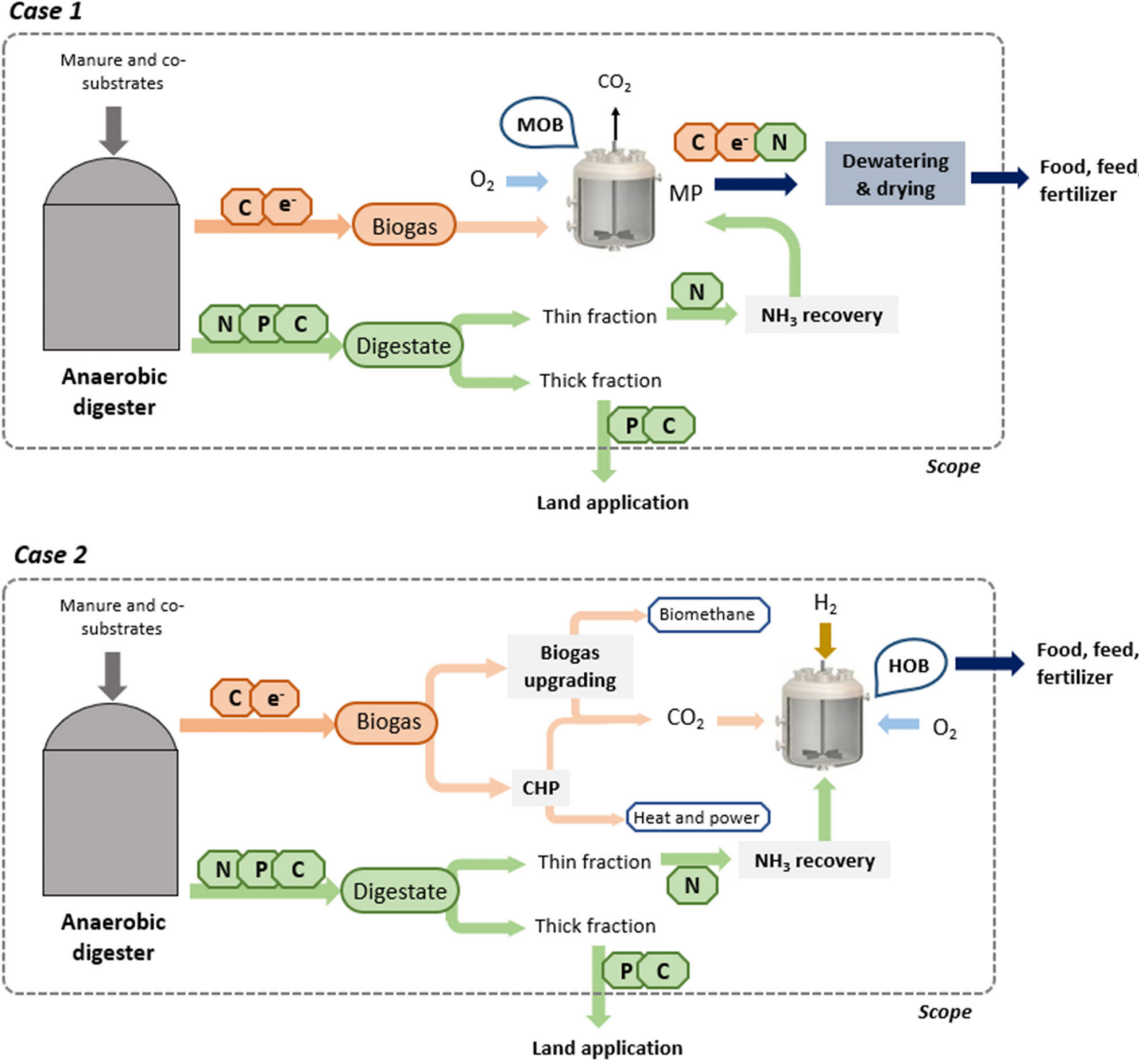
Schematic representation of the two microbial protein production approaches in an anaerobic digestion context. The coloured arrows represent the flows of carbon (C), nitrogen (N), phosphorus (P) and energy (e^‐^) between the different unit technologies (anaerobic digestion, biogas upgrading, biogas combustion in a combined heat and power unit, and microbial protein *via* methane‐oxidizing and hydrogen‐oxidizing bacteria).

The main challenge in this context is the selection of the most cost‐effective MP production pipeline. We determined to which extent different scenarios for on‐site up‐cycling of biogas carbon and recovered mineral nitrogen to microbial protein are economically suitable to be implemented in combination with existing or new AD facilities. To evaluate which MP application could potentially find effective niches for useful application in the AD process, the actual economic performance was calculated, accounting for costs and revenues related to the various production approaches. A model agricultural biogas plant was used as the basis of the calculations. Operational expenditure (OPEX), capital expenditure (CAPEX), potential savings and the revenues from the marketing of the resulting products were determined for the integration of two different MP production routes in a model European AD facility: (i) MOB cultivation on biogas methane and (ii) HOB cultivation on H_2_ with CO_2_ from biogas upgrading or CO_2_ in the flue gases from biogas combustion (Fig. 1). Our evaluation presents the features and economic potential of MP production through valorization of the different building block chemicals available at a digester facility, and could enable the selection of the most appropriate technology for decentralized carbon and nutrient recovery from organic feedstocks through MP.

## Results and discussion

The key concern to push towards nutrient recovery rather than removal from digestate is the economic viability of the proposed recovery scenario. This viability is determined by the total production cost of the product and the market value of the final product(s). Estimations of the costs and revenues associated with the different options for biogas and ammonia upgrading to microbial protein are presented here.

### Biogas as feedstock for microbial protein production by MOB

**Fig. 2:**
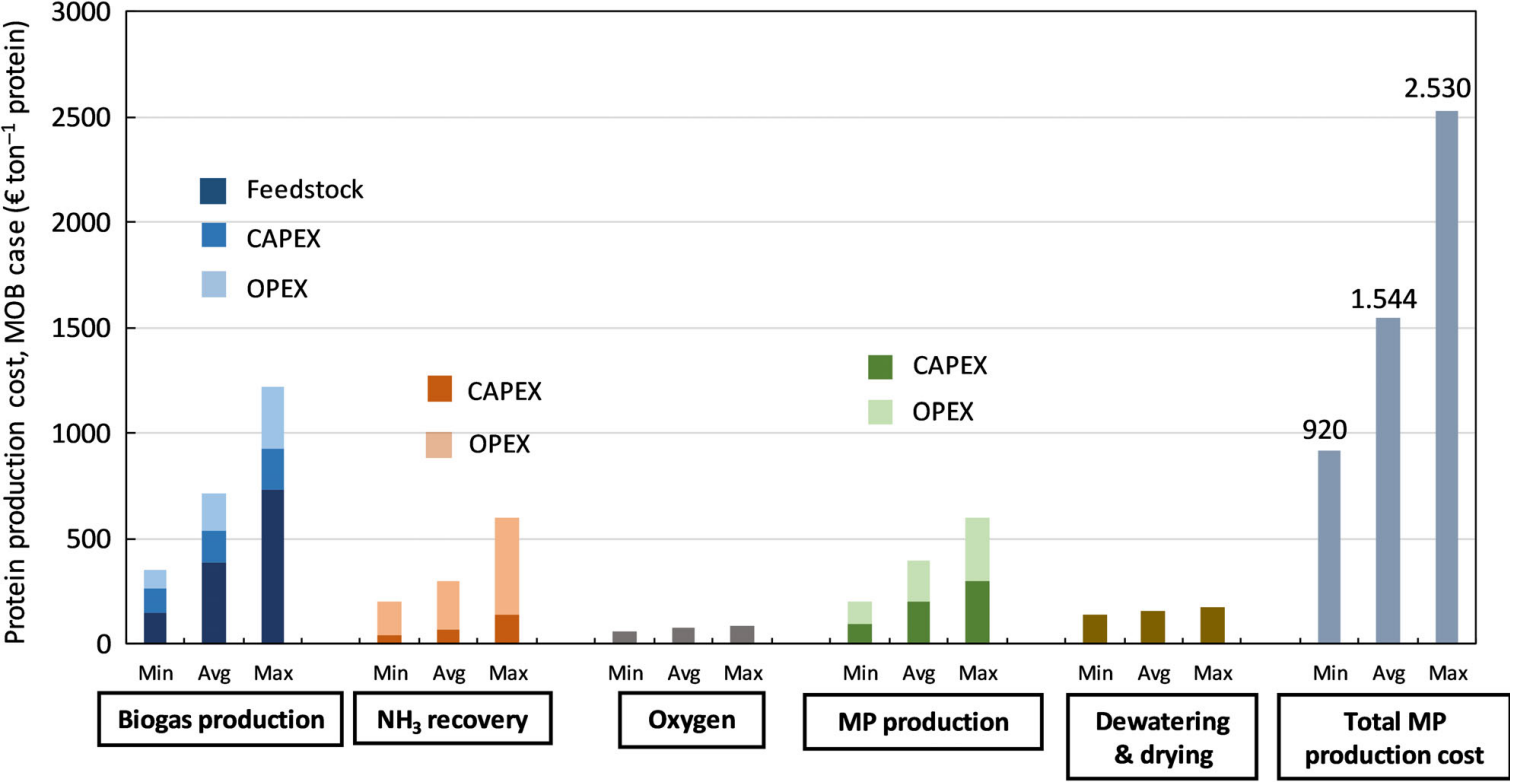
Averaged, minimum and maximum protein production costs using MOB, broken down into components (biogas production, ammonia recovery, oxygen production, dewatering and drying, and the total MP production).

**Fig. 3:**
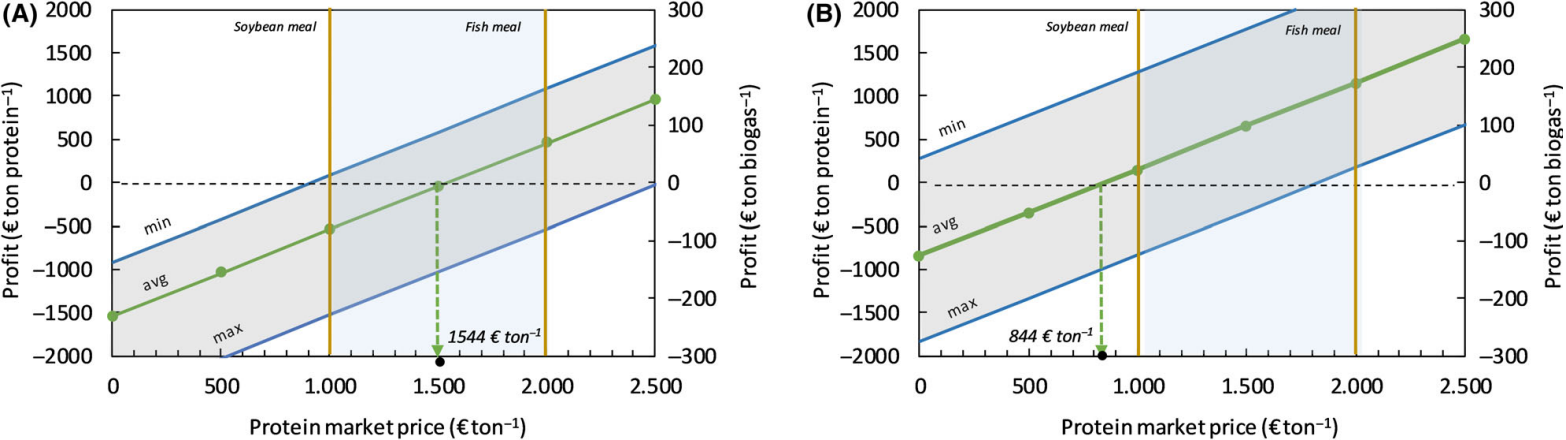
Economic analysis for MP production with CH_4_ as sole carbon and energy source. Profit generated (in € ton^−1^ MP) as a function of the protein market price, not including any financial incentive, for the estimated MP production cost (minimum, average and maximum) without (A) and with avoided costs (B) for nitrogen removal from digestate. The shaded vertical (blue) region represents the variation in current wholesale agro‐based protein price.

The base case production cost of microbial protein obtained from MOB cultivation is estimated at 1544 € per ton crude protein (expressed in 100% dry weight), with 920 € ton protein^−1^ as the minimum and 2531 € ton protein^−1^ as the maximum production costs calculated. A cost breakdown analysis of the total MP production cost is represented in Figure 2. Costs associated with the production/recovery of the building blocks for MOB growth represent 71% of the total base case MP production cost, with 46% for biogas methane, 20% for recovered ammonia and 5% for O_2_, while 19 % can be attributed to CAPEX and OPEX of the MP production unit (293 € ton MP^–1^) and 10 % to dewatering and drying of the wet product (160 € ton MP^−1^). Considering a market price for feed proteins that typically ranges between 1000 € ton^−1^ protein for soybean meal (as the reference vegetable protein for livestock, expressed as protein active substance) and 2000 € ton^−1^ protein for fishmeal (as the reference high‐quality animal protein, expressed as protein active substance), MP can be produced from recovered resources at competitive prices. At present, much still depends on factors relating to the quality demands posed on both the input raw materials (degree of refining) and final product (purity of the product), as well as the downstream processing that is required. The amino acid profile and overall nutritive value of a bacterial meal obtained from MOB growth appeared to be comparable to fishmeal and overall better than soybean meal (Øverland *et al*., 2010) and, it is likely that the produced microbial protein has a market value higher than or at least equal to fishmeal. Market values of protein sources are variable and highly depend on the macroeconomic variables, such as the global demand for livestock protein and the natural gas price for Haber–Bosch ammonia synthesis. As both the global protein demand and pristine ammonia price are expected to increase in the near future (FAO, 2019), MP can become a cost competitive route to produce a substitute for soy and fishmeal for animal feed. Figure 3 shows the impact of a change in MP market price on the profitability of this pathway considering the average MP production cost as well as the minimum and maximum values. The base case, using average‐priced methane and ammonia, suggests that at a protein market price of 1750 € ton^−1^, MP can be produced through the CH_4_:NH_3_ route with a profit around 200 € ton^−1^ MP, corresponding with ∼ 33 € ton^−1^ biogas. Taking into account, the savings from the avoidance of the treatment of the mineral nitrogen present in digestate makes this case much stronger. As the dissipation of reactive nitrogen back to the atmosphere as N_2_ by means of nitrification–denitrification comes at a cost of about 3–4 € per kg NH_3_‐N (Van Hulle *et al*., 2010) and 200 kg NH_3_‐N/ton MP is assimilated during MOB cultivation, some 600–800 € per ton MP can be saved on reactive N removal, as a result of the reduced need for nitrogen removal in the digestate. If MP production is evaluated in the context of local nutrient up‐cycling from digestate, almost for the entire range of protein market prices profit can be made (Fig. 3B). The economic viability of an AD facility that turns its self‐produced methane with recovered ammonia into proteins, thus, seems to be guaranteed, at present costs and revenues, without any legal support. The MP revenues can turn a manure processing facility in a cost neutral (or even profit gaining) installation. With an avoided net cost of 10.95–31.61 € per ton manure processed (De Vrieze *et al*., 2019) (equal to about 548–1581 € ton^−1^ protein produced), MP production seems to be a prime candidate technology to offset the costs associated with manure processing. The reason for this economically justified implementation of MP production technology is twofold. First, MP production could strongly increase the value chain of recovered nitrogen from around 1 € kg^−1^ N for (NH_4_)_2_SO_4_ up to 16.7 € kg^−1^ N for microbial protein. Second, MP production bypasses the low inherent value of methane when energetically valorized on‐site (in a CHP unit) or off‐site (as biomethane in a power plant or car engine), generating more value per ton biogas. As discussed in our previous study, most biogas projects that produce and sell heat and power can only be economically viable with effective and long‐term financial incentives, compensating for the high production costs of biogas/biomethane compared to their market value (Verbeeck *et al*., 2018). For the manure digester under study, governments should give a subsidy of at least 40 € per MWh produced electrical power (equal to 145 € per ton biogas) to realize break‐even operation, considering an electricity wholesale price of 40 € MWh_e_^−1^. Protein production by using methanotrophic bacteria growing on biogas methane would, thus, offer a new business case for AD plants, without dependency on often unstable financial incentives from governments. Our results clearly indicate that through upgrading of low‐value methane and ammonia to protein‐rich microbial biomass, the economic potential of the otherwise often unprofitable exploitation of an AD plant can be strengthened.

It should be mentioned that our economic evaluation does not consider savings on externalized costs of MP production, such as a decreased water consumption, a lower land occupation and decreased nitrogen pollution and greenhouse gas emissions. Some of the key global impacts of MP production were recently discussed by Pikaar and co‐workers (2018b). The same trends were observed in a study that evaluated the environmental impact of FeedKind™ protein, a MP produced from natural gas at commercial scale. The report shows that the water foot print of MP is about 20–140 times lower than fishmeal and soybean meal, respectively, and land use is > 100 times lower compared to soy proteins (Cumberlege *et al*., 2016). Including the externalized environmental costs of the current agro‐production system in the price of protein would result in an allocation of resources that is more efficient for all of society as the MP route is a more rational alternative, able to offer immediate advantages in terms of water and land use (Matassa *et al*., 2016a).

As raw materials represent 66 % of the total cost, the major cost decrease can theoretically be achieved at the level of the digester and the ammonia recovery unit. However, both technologies are already very mature, and the cost decreases that could be expected are limited and more related to scale effects, rather than technological advances. In fact biogas represents, at present, already a relatively inexpensive source of renewable methane for on‐site production, as the consumer price of natural gas for industrial end users is around 440 € ton^−1^ (EU‐28 average price in 2015) (EC, 2016), compared to 326 € ton^−1^ calculated for the base case in this study. This is mainly due to the high transmission and distribution costs of natural gas (see Chapter 2). In contrast, realizing that the gate cost for pristine ammonia is approximately 575 € ton^−1^ NH_3_‐N (Schnitkey, 2018), and the use of recovered nitrogen is, at present, 2–6 times more expensive compared to Haber–Bosch derived NH_3_. As 1 ton proteins can be produced at a cost of 1359 € ton^−1^ protein with freshly synthesized reactive nitrogen (data not shown), nutrient recovery costs, together with avoided removal costs, will be decisive to guarantee the economics of future MP production pipelines. It needs to be recognized that the costs of nitrogen removal *via* stripping/absorption from highly ammonia‐loaded used water streams (> 4 g l^−1^) are in our base case estimated a factor 2 lower than conventional nitrogen dissipation *via* nitrification–denitrification. Above 2 g NH_3_–N L^−1^, commercial stripping installations are able to recover NH_3_ at a cost down to 1000–3000 € ton N^–1^ (Menkveld and Broeders, 2018), while treatment costs of the nitrification–denitrification process are estimated at 3400–4000 € ton^−1^ NH_3_‐N (Van Hulle *et al*., 2010; van Eekert *et al*., 2012). Considering that stripping could remove up to 90 % of the NH_3_ in the liquid fraction, the nitrogen input at the wastewater treatment facility is drastically reduced, and a substantial reduction in costs at these facilities can be achieved. Furthermore, the release of free ammonia by the digester microbiome is so intensive that already in some lab‐scale AD reactors an ammonia stripping unit is directly coupled to the digester as a side loop process to avoid inhibition of the methanogens, due to free NH_3_ toxicity. Next to resource recovery, ammonia stripping could, thus, also allow higher biogas production rates (Siegrist *et al*., 2005; Pedizzi *et al*., 2017).

#### Partial self‐supply of feed on farm scale

Assuming that the full methane flow of 5.16 ton CH_4_ per day is converted to microbial biomass at a biomass yield of 0.76 g CDW g^−1^ CH_4_ (60 % crude protein content) (Matassa *et al*., 2015), this accounts up to a daily protein production potential of 2.4 ton (or 3.9 ton if expressed as cell dry weight). If the microbial biomass is used as additional feed source, and considering that the total protein demand for 1 pig is approximately 45 kg (NRM, 2017), yearly, about 19 500 pigs can be raised with the proteins produced from the carbon and nitrogen contained in manure and liberated by anaerobic digestion. Based on an average cycle time of 166 days, a farm of about 8 864 pigs can be supplied with the MP from the resources generated at the digester that is treating manure from about 24 000 pigs (assuming a daily manure production of 5 kg fresh material per pig per day). The use of on‐site generated methane to locally produce bacterial biomass, thus, offers the farmer the opportunity of partial self‐supply of feed (37 % in this specific case), replacing crop‐based protein in animal feed by MP.

As the yield of soybean is on average 3.11 tons DM per hectare per year (Langemeier and Lunik, 2015), an estimated land footprint of 612 hectares would be required to produce the same amount that can be produced *via* MP in a very compact engineered bioreactor environment, that is 204 m^3^ for the case under study. Assuming a bioreactor height of 30 metres, this comes down to a reactor footprint of just 6.8 m^2^. Besides having a much higher efficiency in land and nutrient use, MP do use water very efficiently, up to 99% reduction in water footprint compared to agricultural‐based production (Cumberlege *et al*., 2016). Implementing a circular approach at digester scale, with the basic components recovered from waste and upgraded into new valuable microbial biomass rich in proteins, thus, offers the opportunity to process manure in a cost‐efficient way, still generating a product that generates profit.

#### Manure requires co‐digestion to achieve the ideal C/N ratio for MP production

For a complete valorization of the ammonia–nitrogen recovered on‐site, methane should be available at a CH_4_:N ratio of 11 kg CH_4_ per kg N [(0.76 ton CDW ton^−1^ CH_4_ x 0.12 ton N ton^−1^ CDW) ^−1^]. Considering that for manure the methane yield relative to available nitrogen is limited, that is typically only in the range of 12 to 18 Nm^3^ methane per ton FM, while nitrogen content can reach> 6 g N L^−1^, the CH_4_:N ratio of manure is too low to allow for a full valorization of the nitrogen present in the digestate. For the digester under study, the defined substrate mixture has a N content of 5.1 kg TKN‐N per ton FM. Accounting for a 75% conversion efficiency of Kjeldahl‐N to NH_4_^+^‐N, a NH_4_^+^ recovery in the liquid digestate of 80% and a 90% NH_3_ stripping efficiency, 2.75 kg NH_4_^+^‐N per ton wet substrate (or 54% of the incoming N load) is extracted from the biomass and, thus, made available for MP production. For the optimal CH_4_:N ratio of 11, this requires a substrate mixture with a methane yield of at least 42 Nm^3^ CH_4_ ton^–1^ FM, highlighting the need to amend manure with co‐substrates to improve the biogas production and obtain a CH_4_:N ratio sufficient for MP production with complete N valorization. For manure, maximum MP production without co‐substrate addition is only possible if an additional electron donor is supplied, either by dosing fossil methane from the natural gas grid or by supply of hydrogen gas to achieve nitrogen assimilation *via* the HOB pathway. The amount of co‐substrate that needs to be mixed with manure is determined by the N content and methane yield of the different substrates. For example, when readily available high strength organic waste streams, like fats or greases with a methane yield up to 800 Nm^3^ per ton FM, are used as co‐substrate (Weiland, 2010), 6 weight % would suffice to achieve the optimal C:N ratio. Opposite, digesters that are limited in nitrogen will need to blend in high N feedstocks or purchase Haber–Bosch NH_3_ to upgrade all available methane

**Fig. 4:**
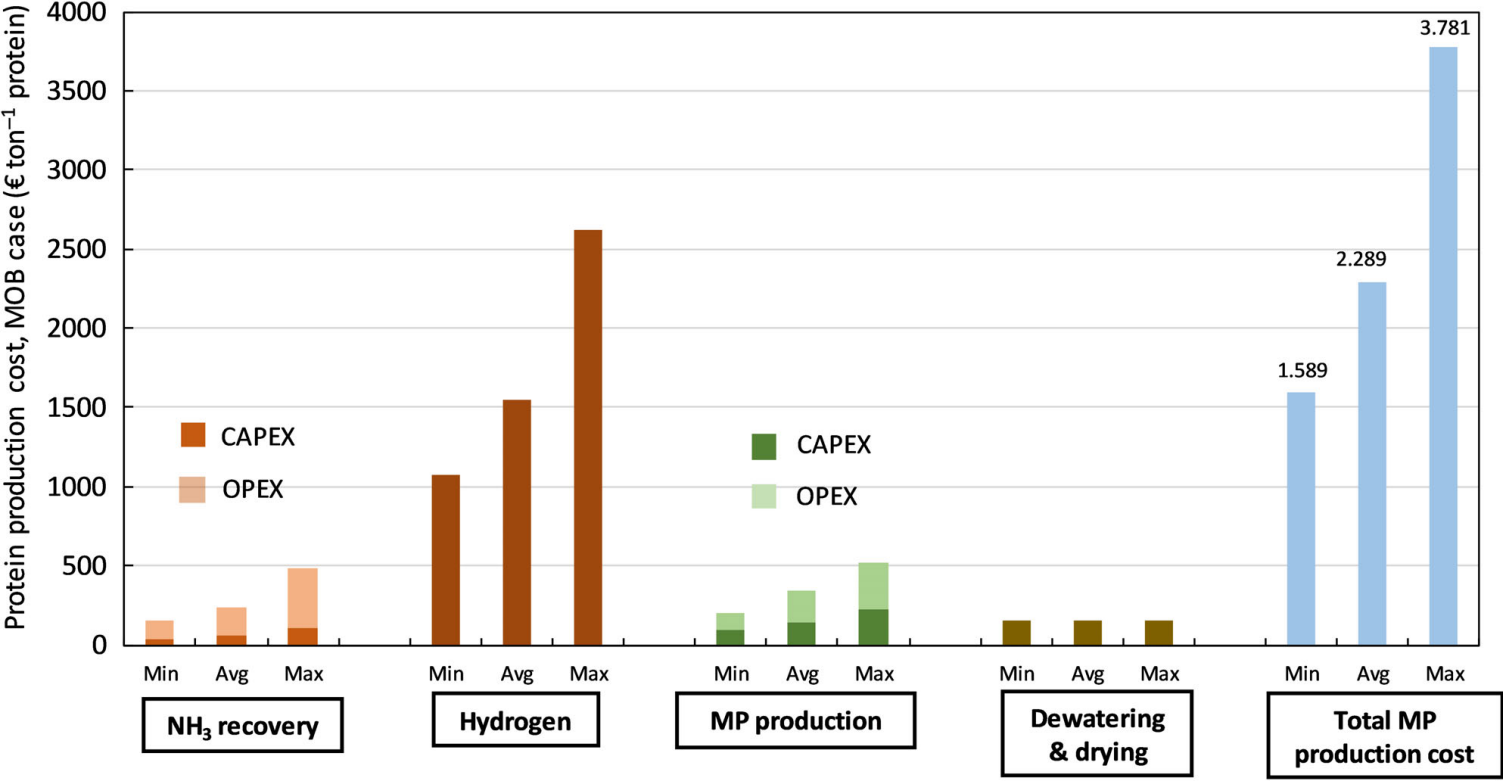
Averaged, minimum and maximum protein production costs using HOB, broken down into components (ammonia recovery, hydrogen production via water electrolysis dewatering and drying, and the total MP production).

#### Resource mining from manure: potential to be import free

Coupling renewable methane generation with the full‐scale production of MP using pure or mixed cultures of methane‐oxidizing bacteria might be the most straightforward approach for MP production in the context of nitrogen and carbon valorization from anaerobic digestion, since MOB cultivation on fossil methane is already well established with several industrial demonstration plants in operation (e.g. Feedkind™ by Calysta and UniProtein™ by UniBio A/S). The large amounts of renewable carbon and recovered nitrogen make manure digesters prime candidate facilities to shortcut the current unbalanced nitrogen cycle. Considering that livestock manure accounts for a nitrogen flow through the EU economy of about 6–9 Mton per year (Foged *et al*., 2012), nitrogen upgrading from anaerobic digestate through MP production processes could produce some 27–40 Mton of microbial biomass, representing 16.2–24.0 Mton crude protein. Currently, the EU imports 20 Mton soybean per year (equal to approximately 9 Mton crude protein) (Schreuder and De Visser, 2015). This means that if we could upgrade 38–56% of the nitrogen from livestock manure to protein, the EU can already be import free, highlighting MP are the prime candidate alternative protein source, surpassing soy and animal meat proteins.

### CO_2_ as carbon feedstock for protein production using HOB

For the H_2_‐CO_2_ route, the profitability of the biogas utilization scenarios, that is power generation or biomethane injection, is not influenced by the production of MP, and CO_2_ is envisaged as an unavoidable product of biogas upgrading/combustion that is fully allocated to the production cost of green electricity or methane (no CO_2_ cost was taken into account for MP production). Although CO_2_ fixating HOB could yield a potential revenue of ~ 160 € per ton protein in carbon credits (at a carbon allowance price of 50 € ton^−1^ CO_2_), no savings are taken into account as CO_2_ emissions from a biogas plant are considered CO_2_ neutral due to their biogenic origin. The HOB fermenter can be considered as a biogas upgrading unit itself, due to its capacity to fix CO_2_ from the biogas. This would eliminate the need for additional technologies, making the biomethane production cheaper. However, these savings are not considered in this assessment as the upgrading potential of a bioreactor is limited.

#### CO_2_ from upgrading biogas to biomethane

**Fig. 5:**
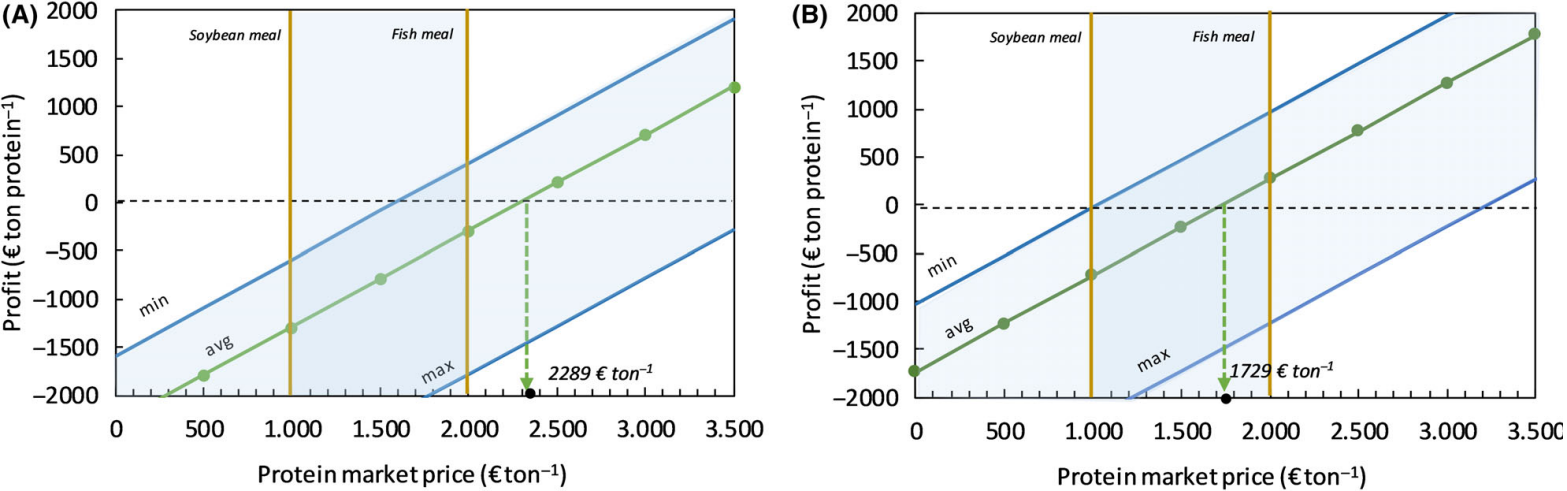
Economic analysis for MP production with H_2_ as energy donor. Profit generated (in € ton^−1^ MP) as a function of the protein market price, not including any financial incentive, for the estimated MP production cost (minimum, average and maximum) without (A) and with avoided costs (B) for nitrogen removal from digestate. The shaded vertical (blue) region represents the variation in current wholesale agro‐based protein price.

With the daily flow of 9.6 ton CO_2_ in the tail gas stream from the upgrading unit, about 2.9 ton crude protein DM can be produced (or 3.9 ton of dry microbial‐based biomass with a crude protein content of 75%), provided that H_2_ is supplied at the required feeding ratio. Production costs of protein by HOB are estimated based on the costs to produce hydrogen gas (and oxygen gas) *via* water electrolysis, recover NH_3_ *via* ammonia stripping, operate the fermenter and dewater/dry the final product. The total base case production cost of 1 ton HOB biomass is estimated at 2289 € ton^−1^ (expressed as dry crude protein) (Fig. 4). Minimum and maximum costs are estimated at 1589 and 3781 € ton MP^−1^, respectively, under the assumptions for extremes made (Table S2). The cost breakdown clearly indicates that hydrogen gas production will be cost decisive. The hydrogen production costs by means of water electrolysis comprise about 67% of the total production costs for H_2_‐based microbial MP. This estimated base case MP production cost was based on a predicted levelized cost of hydrogen of 2.4 € per kg through water electrolysis using renewable energy at a unit price of 44 € per MWh. As recent bids for electricity produced with large‐scale solar photovoltaics have reached prices as low as 30 $ per MWh generated (Haegel *et al*., 2017), it is not unthinkable that these costs will further decrease down to < 2 € per kg H_2_. Considering a mean avoided cost of 3.5 € kg^−1^ N when implementing ammonia recovery instead of nitrogen removal *via* nitrification–denitrification, each ton MP produced saves about 560 € on wastewater treatment costs, making the economics look differently (break‐even point at 1729 € ton^−1^ MP, Fig. 5B).

Per kg protein produced, cells assimilate about 0.16 kg NH_3_‐N, leading to a gross daily uptake of 463 kg N, equal to 97.5% of the nitrogen that could be extracted from the liquid digestate *via* stripping. The C:N of the feedstock mixture is, thus, sufficient for a full conversion of CO_2_‐C and NH_3_‐N.

Current practice for N recovery is mainly air or steam stripping, which is energy intensive, that is 3.9 to 28.2 kWh kg N^−1^ depending on the scale of the plant (Gulyas *et al*., 2014), and requires caustic and acid dosage for stripping and scrubbing respectively. Recently, a proof of concept for NH_3_ extraction from urine through electrochemical stripping was put forward as an energy‐efficient way to produce a gas mix that was used for microbial protein production by HOB at less than 10 kWh kg^−1^ N when H_2_ energy is considered. This process, which can be fully driven by renewable power, brings the 4 key building blocks for growth of HOB from 1 process: H_2_ and NH_3_ from the cathode and O_2_ and CO_2_ (originating from the urea hydrolysis product HCO_3_^−^) from the anode (Christiaens *et al*., 2017). Moreover, *via* the introduction of a membrane to assist the electrochemical stripping, the risks for cross‐over of microorganisms and trace contaminants into the nitrogen product flow was minimized (Christiaens *et al*., 2019).

It needs to be recognized that due to the lower growth rate of HOB relative to MOB and the lower solubility of H_2_ over CH_4_, more effort is needed to achieve a high intensive H_2_ based protein production. The low solubility of H_2_ and CH_4_ is typically overcome by engineering bioreactor systems that are designed with the specific purpose to achieve very high volumetric gas–liquid mass transfer rates through a combination of increased head space pressure, intense mixing and fine bubble sparging. Full‐scale bioreactor systems that rely on CH_4_, CO and H_2_ as carbon and/or energy source are currently realized by several companies active in MP (UniBio, Calysta) as well as ethanol production (LanzaTech).

#### CO_2_ from CHP unit

For the biogas plant under study, combustion of the daily biogas flow generates 23.7 ton of CO_2_. Without limitations on the availability of the other building blocks for HOB growth, about 7.2 ton protein per day can be produced fixing the CO_2_ in the combustion gases and assimilating about 1.15 ton NH_3_‐N per day. With this production capacity, some 26 000 pigs can be fed daily. However, realizing that nitrogen is the limiting factor in this scenario, that is only 475 kg recovered NH_3_‐N available, a maximum of 3.0 ton protein can be produced daily with the nutrients available on‐site. Additional imports of nitrogen of the order of 675 kg per day are, thus, necessary if all available carbon on‐site is targeted for MP production.

The overall viability of the biogas plant was evaluated for this case as well, taking into account costs and revenues associated with CHP production. Total cost following this CHP‐MP route is estimated at 605 € ton^−1^ biogas. Revenues from selling both electricity at 40 € MWh_e_^−1^ and protein‐rich biomass at 1750 € ton^−1^ MP are around 500 € ton^−1^ biogas, while avoided costs for N removal are about 109 € ton^−1^ biogas. Revenues and savings from MP could, thus, compensate the financial losses from CHP production, enabling a cost‐efficient treatment of manure and organic waste through anaerobic digestion and MP production. In conclusion, the MP production *via* the NH_3_‐H_2_ route is only economically viable when production costs are assumed to be minimal and savings through nitrogen upgrading are taken into account. Further technological advances to bring down the cost might offer perspectives to increase the cost competitiveness.

### Future perspectives

#### Complementary hydrogen and methane platforms

More than being self‐excluding, the methane and the hydrogen gas platforms can be seen as complementary, depending on the availability of each resource on‐site and the value/cost of renewable energy. The MP production from a mixture of methane and hydrogen opens the potential to consider a system that can valorize all gaseous carbon available at a biogas plant. This would imply the collaboration of two aerobic populations, MOB and HOB, in one engineered bioreactor environment. For the case in the study, 5.3 ton MP per day can be produced from the total carbon flow if an additional 460 kg N per day is purchased.

The fact that MOB are well‐studied microorganisms that have been implemented in full‐scale production reactors is a strong asset of this technology platform. When compared with hydrogen‐oxidizing bacteria, methanotrophs offer the benefit that they can be set to work directly on renewable methane without the need for additional energy input. However, relative to HOB, they possess a lower biomass yield, lower growth rates and lower protein levels (Matassa *et al*., 2016b).

There is even a potential for MP production from waste organics, such as carboxylic acids that are generated upon fast anaerobic treatment of organic streams, like slaughterhouse wastewater, although this entails that more attention will be needed for avoiding waste materials crossing over into the product. Emerging as microbial protein are the purple non‐sulfur bacteria that require infrared light and an organic substrate to grow (Hülsen *et al*., 2014), although these come with the evident drawback of needing a photo‐bioreactor. Recently, the use of protein‐rich biomass as slow‐release organic nitrogen fertilizer has been put forward as a novel outcome of MP. Key benefit of producing fertilizer over the MP‐based production of human food and animal feed lies in the fact that processes conditions for non‐food applications are less strict in terms of hygienization, sterilization, composition and dry solid content of the final product (Pikaar *et al*., 2018a). In this perspective, one could look into the option to directly grow MP in the (liquid) digestate, taking up residual carbon and mineral nitrogen from the medium without the need to recover the nutrients prior to MP cultivation. Realizing that stripping and assimilation are both not 100% efficient, there is still an amount of NH_3_‐N that ends up in wastewater treatment plant. To be able to operate in a full recovery mode (without polishing in a nitrification–denitrification step), the production of MP for fertilizer applications through the assimilation of the residual reactive nitrogen is an interesting approach.

#### What is needed to drive implementation of MP at biogas plants?

The strong incentives decarbonization and renewable energy targets drive the valorization of biogas as a local and renewable energy source, either for on‐site CHP production, or *via* injection of upgraded biomethane in the natural gas grid. As long as these ‘green’ feed‐in premiums generate positive business cases for biogas projects, it will be hard to convince AD owners to valorize the methane in a different way, and particularly to consider making major capital investments. However, there is a second carbon feedstock available at the facility that is, at present, in many cases not valorized: CO_2_. Either the CO_2_ produced by upgrading of biogas to biomethane, or the CO_2_ emissions from the combustion of biogas can be exploited as carbon feedstock for protein production using H_2_‐oxidizing bacteria.

It remains questionable whether farmers are willing to up‐cycle carbon and nutrients into edible MP products and replace a part of their crop‐based animal feed protein demand by self‐produced MP. A successful and widespread adoption of the MP biotech platform at biogas facilities is, even under a proven economic profitable plant operation taken into account the revenue from the avoidance of the treatment of the mineral nitrogen, prone to cultural factors in farm management, a lacking official legal recognition and the widespread public acceptance of microbial‐derived products as feed and food additive. Labels that clearly indicate to consumers that meats are produced with a lower environmental footprint could assist in market uptake, similar to labels such as 'organic'. It could even be considered that legislators put a cap on acceptable GHG and mineral nutrient emissions per unit meat protein to stimulate alternative sourcing. In this way, the high externalized environmental costs of the current conventional agricultural‐based supply routes for animal‐based proteins would be made clear to the public, playing in favour of establishing a mindset more open to acceptance of alternative protein sources with a lower environmental impact. However, the market entrance of MP as main protein additive in livestock production and aquaculture is probably less a concern compared to the direct consumption as human food as the product quality and taste of the meat will not be affected, and consumers are not directly in touch with the microbial‐based product.

Obviously, safety and quality of the edible MP products must be guaranteed in order to allow a successful adoption of microbial‐based products, for sure when produced from carbon and nutrients recovered from organic waste such as livestock manure. In this light, it is essential to sterilize the MP product and to provide safety barriers between the waste stream and the final product to avoid cross‐over of potential opportunistic pathogens or harmful contaminants to the final product (*e.g*. membranes).

## Conclusion

To ensure that both products of anaerobic digestion, that is biogas and digestate, are utilized to their full potential as renewable sources of raw materials, new valorization pipelines need to be implemented into the current AD process schemes. At present, products deriving from digestate achieve a low market value and recovery costs cannot be offset by the revenues. Nutrient recovery processes like ammonia stripping or struvite production, however, might represent the starting point of an entire new biorefinery concept in which microorganisms grow on renewable carbon sources and recovered reactive nitrogen while producing protein‐rich microbial biomass (known as microbial proteins). The already well‐established methane‐oxidizing bacteria represent a promising technology to upgrade low‐value methane and nitrogen to a product than can be used as an alternative high‐quality food/feed protein source, surpassing the conventional agro‐based protein generation. The technology for microbial protein production in the framework of an anaerobic digester facility that turns its self‐produced methane with recovered ammonia into proteins is of micro‐economic interest, as this pipeline offers a better return on investment than burning biogas and the use of digestate products for land application. The MP revenues can turn a manure processing facility in a cost neutral (or even profit gaining) installation. For the NH_3_‐H_2_ case, calculations show that this route is of interest if the protein value equals the value of high‐quality agro‐based proteins like fishmeal and if the avoided costs for N removal are taken into consideration. As hydrogen production costs are expected to decrease further, the process will be of higher economic relevance in the future and will, thus, enable maximal utilization of carbon processed through anaerobic digesters. Overall, this study presents an interesting approach to partially shortcut the nitrogen cycle at the scale of a digester facility by direct introduction of MP as feed for animals.

## Experimental procedures

Two different scenarios were designed for the recovery of carbon and nitrogen from biogas and digestate respectively. Each case has been studied for a model agricultural AD plant with a nominal raw biogas flow of 500 Nm^3^ per hour, 60 vol.% CH_4_, and a digester N load of 36.7 kg TKN‐N per hour (5.1 kg TKN‐N ton^−1^ fresh material), that is:

- Protein production based on the methane in the biogas by MOB *(CASE 1,* Fig. 1 *)*.
- Protein production based on the CO_2_ from biogas upgrading to biomethane or from biogas combustion by extra energy input in the form of hydrogen gas and using HOB *(CASE 2,* Fig. 1 *)*.

Each scenario contained a different combination of processes, depending on the carbon, energy and nutrient source for MP production and the integration within the AD facility. Performance was evaluated based on an extensive literature review and steady‐state mass balancing of the different unit operations to determine biogas production, nitrogen release, ammonia recovery efficiency and MP production potential for each case. The costs of the input materials as well as capital and operational costs were estimated based on available data in literature. The economic viability of each scenario was assessed in terms of protein benefits and input costs for MP production. To account for the variability in cost estimations that can be found in the literature, the minimum and maximum costs are calculated as well (Table S2). The methodology and main assumptions regarding costs and revenues are summarized in the following sections.

### Input side: raw materials and costs

#### Biogas

We assumed a model mesophilic farm‐based digester fed with an agricultural feedstock mixture dominated by pig manure (70 % of the total fresh material input, wet weight). The manure was collected from several pig breeding facilities and processed in a central AD installation together with three co‐substrates available in close proximity of the digester: agricultural residues (representing 10 w.% of the fresh material going into the digester), food waste (food processing residues, 10 w.%) and energy crops (maize silage, 10 w.%) were selected as co‐substrates to increase the biogas yield and operational stability. The substrate mixture has a weighted average methane yield of 42 m^3^ methane per ton fresh material and is calculated based on the methane yield of the different feedstocks (supporting data in Table S1). On a dry solid basis, the manure represented 41%, energy maize and agricultural residues both 22%, and the food processing residues 15% of the total solids load going into the digesters.

As a reference technology for nutrient recovery from the raw digestate, a centrifugal separation into a liquid and solid fraction was selected, after which the solid fraction, rich in slowly digestible organic matter and organically bound nutrients, was used for composting (and thus land use), while the nitrogen‐rich aqueous phase was subjected to gas stripping and subsequent absorption in a sulfuric acid scrubbing solution to form ammonium sulfate (Vaneeckhaute *et al*., 2017). It is assumed that direct local land application of the recovered inorganic fertilizer is not feasible since land application limits are stringent and do not allow for a full reuse of the nutrients recovered from the manure and the co‐substrates. The main technical design data are listed in Table S1 (in Supporting Information) and characterize the digester’s supply chain, from the feedstock up to the quantity of biogas produced and ammonia–nitrogen liberated.

The average total capital investment of equipment and construction is set to correspond with an investment for biogas production of 4000 € Nm^−3^ h^−1^ installed biogas capacity (IRENA, 2013). OPEX costs for the digester were calculated based on a fixed percentage of the CAPEX (7.5 % of the investment sum on a yearly basis), including electricity and chemicals consumption, maintenance and labour (Verbeeck *et al*., 2018). The feedstock mixture is assumed to have a fixed cost of 5.28 € ton^−1^ fresh material, transportation included (pig manure and crop residues were assumed to have no cost) (Verbeeck *et al*., 2018). The average specific raw biogas production cost for the agricultural digester under study was estimated at 115 € ton^−1^ biogas or 326 € ton^−1^ methane (Table S2). Estimations of minimum and maximum costs are included in Table S2.

#### Ammonia

The overall cost to recover 1 ton NH_3_‐N by means of conventional air stripping/absorption ranges from 1000 to 3000 € ton^−1^ NH_3_‐N with ammonium sulfate as the recovered product. Considering the high N‐concentration in liquid fraction of the digested manure (> 4 g l^−1^), an average recovery cost of 1500 € ton^−1^ NH_3_‐N was assumed as base case, with 1000 and 3000 € ton^−1^ NH_3_‐N for the extreme cases (Menkveld and Broeders, 2018). The percentage of N present in the feedstock that ends up in the liquid fraction was assumed to be 80 % (Weiland, 2010). The removal efficiency for NH_3_‐N *via* stripping was set at 90% (Menkveld and Broeders, 2018). The uptake efficiency of mineral nitrogen by the bacterial culture is assumed to be 100% as reported by Matassa *et al*. (2016).

#### CO_2_

Since CO_2_ is inherently linked to the biogas production, we did not allocate costs to the CO_2_ feed. Upgrading and injection as well as CHP costs are allocated to the production of biomethane or power.

#### Hydrogen gas

At present, the costs for hydrogen production by means of PEM electrolysis including CAPEX and OPEX are estimated at 4400 € ton^−1^ H_2_ (based on an electricity price of 44 € MWh^−1^). Future predicted levelized costs for hydrogen production was set at 2600 € ton^−1^ H_2_ (Ayers *et al*., 2010). It is forecasted that renewable power costs will reduce to 30 € MWh^−1^ by 2020–2025, and even down to 10 € MWh^−1^ by 2030–2040 (Fraunhofer, 2015), bringing down the electricity cost to < 2000 € ton^−1^ H_2_.

#### Oxygen gas

Oxygen limitation is prevented by aerating the fermenter with pure oxygen instead of air. It has been reported by Bélanger and colleagues that pure oxygen injection permitted a longer exponential phase in high cell density fermentations for protein production (2004). The oxygen needed for MOB cultivation would have to be produced in an additional process (i.e. *via* cryogenic separation or pressure swing adsorption). The mean cost for generation of industrial grade oxygen is estimated at 30 € ton^−1^ O_2_ (Allam, 2009). For the HOB case, oxygen is co‐produced along with hydrogen in the electrolysis process. Given the fact that oxygen is not a limiting raw material in the produced quantities in relation to hydrogen gas (∼ 8 kg O_2_ per kg H_2_ produced during electrolysis of water), the cost for O_2_ is covered by the cost for H_2_. An oxygen requirement for MOB and HOB production of 2.50 and 2.05 ton O_2_ per ton MP is taken into account respectively (Table S4).

### Microbial protein production and drying: Opex and Capex

The total capital investment of equipment and construction is set to correspond with an investment of 5000 € m^−3^ installed reactor capacity (estimation based on Peters *et al*., 2003 and pers. communication). A depreciation period of 20 years with an interest rate of 5% was assumed. A volumetric production rate for MOB and HOB of, respectively, 0.48 and 0.31 kg protein per m^3^ of reactor per hour was used as the basis of the required reactor volume (Equation 1). As a full conversion of the substrates is targeted, MP production rates are set at 20 % of the maximum rates reported in literature (that typically are obtained at high substrate loading rates that do not aim to achieve 100 % conversion) (see Table S3 for these maximum reported productivities).

(1) $$\text{Required}\;\text{reactor}\;\text{volume}=\frac{\text{Microbial}\;\text{protein}\;\text{production}\;\text{rate}\;(\text{ton}\;\text{per}\;h)}{\text{Volumetric}\;\text{production}\;\text{rate}\;(\text{ton}\;\text{per}\;m^{3}\;\text{per}\;h)}$$

The OPEX contribution to the total cost was set at a fixed sum of 200 € ton^−1^ MP, including utilities, labour and supervision, overhead, and maintenance. Raw material costs other than CO_2_, CH_4_, O_2_, H_2_ and NH_3_ (like phosphorus, trace elements, micro nutrients and pH control chemicals) are included in the OPEX. Separation, sterilization and drying costs were set at 160 € ton^−1^ MP, based on the calculations performed in a recent study of Pikaar *et al*. (2018b). This is the sum of the energy costs related to water removal by centrifugation (leaving a product with around 25% DM content) and spray‐drying with integrated fluidized bed technology up to a dry solids content in the final product of 100 %. Assumptions made for the extreme cases are listed in Table S2.

### Output side: protein and revenues

The assumed yields, protein content and stoichiometry of MOB and HOB cultivation are listed in Tables S3 and S4, and form the basis of the MP production taking into account the amounts of recovered feedstocks for bacterial growth. MOB and HOB biomass was assumed to consist of 12 wt.% nitrogen (Matassa *et al*., 2016b). An average market value of 1750 € ton^−1^ protein of the produced microbial biomass will be taken into account in this study as its protein and amino acid composition is comparable to that of fishmeal (which is worth in between 1500 and 2300 € ton^– 1^ protein) (RaboResearch Food and Agribusiness, 2019). Moreover, it is known that under stress conditions, especially under oxygen or nutrient limiting conditions, the microbial cells are able to accumulate polyhydroxybutyrate (PHB), a biopolymer used as energy storage by bacteria (Khosravi‐Darani et al., 2013). This PHB is of special value for enhanced feeds as PHB are regarded as prebiotic feed additive and microbial control agent when used in the diet of different aquaculture species (De Schryver *et al*., 2010). This product could bring additional nutritional value to the produced microbial cells, but this added value is not taken into account in the economic evaluation.

Process profitability is estimated considering OPEX, CAPEX, feedstock cost and protein market price. The profit is expressed per ton biogas and per ton MP (expressed as 100% protein crude content), taking into account the different input streams. To account for variability and uncertainty regarding cost estimations, a parametric analysis was performed, providing details on the impact of operational cost and revenues on the economy of the facility under study.

## Conflict of interest

All authors declare no conflicts of interest.

## Supporting information


**Table S1** **.** Main technical design data and general assumptions.
**Table S2** **.** Estimation of the production cost of input materials for MP production at digester scale.
**Table S3** **.** Comparison between methane‐oxidizing bacteria and hydrogen‐oxidizing bacteria used in MP production.
**Table S4** **.** Input feed to produce 1 ton MP via HOB *vs*. MOB.
