# Supplementary material for: Assessing the potential for up‐cycling recovered resources from anaerobic digestion through microbial protein production
Source: Microb Biotechnol. 2020 Jun 11;14(3):897–910. doi: 10.1111/1751-7915.13600 (PMC8085915; doi:10.1111/1751-7915.13600)
Supplement: Supplementary file 1 — Table S1 . Main technical design data and general assumptions. Table S2 . Estimation of the production cost of input materials for MP production at digester scale. Table S3 . Comparison between methane‐oxidizing bacteria and hydrogen‐oxidizing bacteria used in MP production. Table S4 . Input feed to produce 1 ton MP via HOB vs. MOB. [file MBT2-14-897-s001.pdf]

Supplementary information

Assessing the potential for up-cycling recovered resources from anaerobic digestion through microbial protein production

Kristof Verbeeck[1,2], Jo De Vrieze[1], Ilje Pikaar[3], Willy Verstraete[4], Korneel Rabaey[1,5]✉

[1] Center for Microbial Ecology & Technology (CMET), Ghent University, Coupure Links 653, B-9000 Gent, Belgium

[2] Current affiliation: ArcelorMittal Belgium, John F. Kennedylaan 51, 9042 Gent, Belgium

[3] Advanced Water Management Centre (AWMC), The University of Queensland, QLD 4072, Australia

[4] Avecom NV, Industrieweg 122P, B-9032, Wondelgem, Belgium

[5] CAPTURE, www.capture-resources.be

✉ Corresponding author: Korneel Rabaey; Ghent University; Faculty of Bioscience Engineering; Center for Microbial Ecology and Technology (CMET); Coupure Links 653; B-9000 Gent, Belgium; phone: +32 (0)9 264 59 76; fax: +32 (0)9 264 62 48; E-mail: Korneel.Rabaey@UGent.be.

List of Tables

Table S1. Main technical design data and general assumptions.

**Feedstock mix**	**Biogas yield [a] (Nm^{-3} biogas kg^{-1} FM)**	**N content [b] (g TKN-N kg $^{-1}$ FM)**	**w.% of feedstock (on FM basis)**
Maize silage	210	2.07	10 %
Crops residues	100	0.83	10 %
Food waste	190	4.06	10 %
Pig manure	28	6.30	70 %
Weighted average of the mix	*69.4*	*5.11*	
Biogas production rate (Nm^3 h^{-1} / ton day^{-1})	500 / 14.75		
Biomass input (ton FM day^{-1})	172		
Total solids input (ton TS day^{-1}) [c]	25.4		
**Nitrogen mass balance**		*Recovery efficiency*	
TKN-N loading rate to AD (kg N day^{-1})	880		
NH_4^+-N release rate in AD (kg N day^{-1})	660	75 % [d]	
NH_4^+-N recovery in liquid digestate (kg N day^{-1})	528	80 % [a]	
NH_4^+-N recovery through stripping (kg N day^{-1})	475	90 % [e]	
Energy density methane (kWh Nm^{-3}), LHV	10.85		
CH_4 content raw biogas (vol.%)	60 %		
Energy density biogas (kWh Nm^{-3})	6.50		
Mass density biogas (kg Nm^{-3})	1.22		
Energy density biogas (MWh ton$^{-1}_{biogas}$)	5.33		

**FM = fresh material*

[a] *(Weiland, 2010)*

[b] *(Drosg et al., 2015; Pintucci et al., 2017)*

[c] *TS content of substrates: manure(8.7 %), corn & crop residues (33 %) and food waste (20.8%) (Pintucci et al., 2017):*

[d] *(De Vrieze et al., 2019)*

[e] *(Menkveld & Broeders, 2018)*

Table S2. Estimation of the production cost of input materials for MP production at digester scale.

1. **Biogas production cost**	*Minimum*	***Average***	*Maximum*
Capital investment (€)[a]	1 500 00	**2 000 000**	2 500 00
CAPEX (€ ton^{-1} biogas)[b]	*18*	***25***	*31*
OPEX (€ year^{-1})[c]	75 000	**150 000**	250 000
OPEX (€ ton^{-1} biogas)	*14*	***28***	*46*
Feedstock cost (€ ton^{-1} FM)[d]	2	**5.28**	10
Feedstock cost (€ ton^{-1} biogas)	*23*	***62***	*117*
Production cost (€ ton^{-1} biogas)	*56*	***115***	*194*
Production cost (€ ton^{-1} methane)[e]	*156*	***326***	*550*

[a] Assumed investment: 3000 (Min), 4000 (Avg) and 5000 (Lechtenböhmer et al.) € Nm^{-3} h^{-1} installed biogas capacity. Investment without investment subsidy or support.
[b] Depreciation period of 20 years, 3 % interest rate.

[c] Assuming 5 % (Min), 7.5% (Avg) and 10 % (Lechtenböhmer et al.) of the total investment per year.

[d] Maize silage was assumed to have a feedstock cost of 32.8 € ton^{-1} FM and food waste a cost of 20 € ton^{-1} FM (including transportation). Agricultural residues and manure were assumed to come in for free.
[e] Assumed methane content is 60 vol.%.

2. **Ammonia recovery cost**	*Minimum*	***Average***	*Maximum*
CAPEX (€ ton^{-1} N recovered)	*230*	***345***	*460*
OPEX (€ ton^{-1} N recovered)[a]	*770*	***1 155***	*1 540*
Total N recovery cost (€ ton^{-1} N recovered)	*1 000*	***1 500***	*2 000*

[a] Based on an influent stream containing a NH_3-N concentration of > 4 g L^{-1}.

3. **Hydrogen production cost**	*Minimum*	***Average***	*Maximum*
Total production cost (€ ton^{-1} H_2)	*1 800 [b]*	***2 600 [a]***	*4 400 [c]*

[a] Future predicted levelized cost for hydrogen production using PEM electrolysis at an electricity price of 44 € per MWh. (Ayers et al., 2010)
[b] (Gökçek, 2010)

[c] Current cost for hydrogen production using PEM electrolysis at an electricity price of 44 € per MWh. (Ayers et al., 2010)

4. **Oxygen production cost[a]**	*Minimum*	***Average***	*Maximum*
	26	***30***	*34*

[a] Only for MOB case, as for the HOB case oxygen is produced via electrolysis, and costs are allocated to hydrogen.

5. **Microbial protein production**	*Minimum*	***Average***	*Maximum*
Capital investment (€ m^{-3} $_{reactor}$)	*3 000*	***5 000***	*8 000*
OPEX (€ ton^{-1} MP)	*100*	***200***	*300*
6. ***Dewatering and drying***	*Minimum*	***Average***	*Maximum*
Centrifugation (€ ton MP^{-1})	-	***70***	-
Spray drying (€ ton MP^{-1})	-	***90***	-

[b] (Pikaar et al., 2018b)

Table S3. Comparison between methane-oxidizing bacteria and hydrogen-oxidizing bacteria used in MP production.

	Methane-oxidizing bacteria	*Hydrogen-oxidizing bacteria*
Cell yield (g CDW g^{-1} COD)	0.19 [a]	0.28 [b]
Protein content (% Protein on CDW)	60 % [c]	75 % [d]
Volumetric production rate (kg CDW m^{-3} h^{-1})	4 [e]	2.28 [f]
N content (% N on CDW)	12 %	12%

[a] (Higgins et al., 1981)
[b] (Ishizaki & Tanaka, 1990)
[c] (Yazdian et al., 2005)
[d] (Volova & Barashkov, 2010)
[e] (Ritala et al., 2017)
[f] (Tanaka et al., 1995)

Table S4. Input feed to produce 1 ton MP via HOB *vs.* MOB.

	Feed (ton)					Product (ton)
Case	CH_4	CO_2	NH_3	H_2	O_2	Microbial protein
MOB	2.20	-	0.20	-	2.50	1
HOB	-	3.31	0.16	0.79	2.05	1

References

Ayers, K.E., Anderson, E.B., Capuano, C., Carter, B., Dalton, L., Hanlon, G., Manco, J., Niedzwiecki, M. (2010) Research advances towards low cost, high efficiency PEM electrolysis. *ECS Transactions*, **33**(1), 3-15.

De Vrieze, J., Colica, G., Pintucci, C., Sarli, J., Pedizzi, C., Willeghems, G., Bral, A., Varga, S., Prat, D., Peng, L., Spiller, M., Buysse, J., Colsen, J., Benito, O., Carballa, M., Vlaeminck, S.E. (2019) Resource recovery from pig manure via an integrated approach: A technical and economic assessment for full-scale applications. *Bioresource Technology*, **272**, 582-593.

Drosg, B., Fuchs, W., Al Seadi, T., Madsen, M., Linke, B. (2015) Nutrient recovery by biogas digestate processing. *IEA Bioenergy*, **2015**, 711.

Gökçek, M. (2010) Hydrogen generation from small-scale wind-powered electrolysis system in different power matching modes. *International Journal of Hydrogen Energy*, **35**(19), 10050-10059.

Higgins, I.J., Best, D.J., Hammond, R.C., Scott, D. (1981) Methane-oxidating microorganisms. *Microbiological Reviews*, **45**(4), 556-590.

Ishizaki, A., Tanaka, K. (1990) Batch culture of Alcaligenes eutrophus ATCC 17697T using recycled gas closed circuit culture system. *Journal of Fermentation and Bioengineering*, **69**(3), 170-174.

Lechtenböhmer, S., Nilsson, L.J., Åhman, M., Schneider, C. (2016) Decarbonising the energy intensive basic materials industry through electrification – Implications for future EU electricity demand. *Energy*, **115**, 1623-1631.

Menkveld, H.W.H., Broeders, E. (2018) Recovery of ammonia from digestate as fertilizer. *Water Practice and Technology*, **13**(2), 382-387.

Pintucci, C., Carballa, M., Varga, S., Sarli, J., Peng, L., Bousek, J., Pedizzi, C., Ruscalleda, M., Tarragó, E., Prat, D. 2017. The ManureEcoMine pilot installation: advanced integration of technologies for the management of organics and nutrients in livestock waste. *Water Science and Technology*, **75**(6), 1281-1293.

Pikaar, I., Matassa, S., Bodirsky, B.L., Weindl, I., Humpenöder, F., Rabaey, K., Boon, N., Bruschi, M., Yuan, Z., van Zanten, H., Herrero, M., Verstraete, W., Popp, A. (2018). Decoupling Livestock from Land Use through Industrial Feed Production Pathways. *Environmental Science & Technology*, **52**(13), 7351-7359.

Ritala, A., Häkkinen, S.T., Toivari, M., Wiebe, M.G. (2017) Single cell protein—state-of-the-art, industrial landscape and patents 2001–2016. *Frontiers in microbiology*, **8**, 2009.

Tanaka, K., Ishizaki, A., Kanamaru, T., Kawano, T. 1995. Production of poly (d-3-hydroxybutyrate) from CO2, H2, and O2 by high cell density autotrophic cultivation of Alcaligenes eutrophus. *Biotechnology and bioengineering*, **45**(3), 268-275.

Volova, T.G., Barashkov, V.A. (2010) Characteristics of proteins synthesized by hydrogen-oxidizing microorganisms. *Applied Biochemistry and Microbiology*, **46**(6), 574-579.

Weiland, P. (2010) Biogas production: current state and perspectives. *Applied microbiology and biotechnology*, **85**(4), 849-860.

Yazdian, F., Hajizadeh, S., Shojaosadati, S.A., Khalilzadeh, R., Jahanshahi, M., Nosrati, M. (2005) Production of single cell protein from natural gas: Parameter optimization and RNA evaluation. *Iran. J. Biotechnol.*, **3**(4), 235-242.
